# Network Mapping of Time to Antithrombotic Therapy Among Patients With Ischemic Stroke and Transient Ischemic Attack (TIA)

**DOI:** 10.3389/fneur.2021.651869

**Published:** 2021-06-07

**Authors:** Thanh G. Phan, Benjamin Clissold, Shaloo Singhal, John Van Ly, Andy Lim, Jason Vuong, Stella Ho, Chelsea Matley, Talvika Kooblal, Henry Ma

**Affiliations:** ^1^Stroke & Aging Research, Department of Medicine, School of Clinical Sciences at Monash Health, Monash University, Clayton, VIC, Australia; ^2^Department of Neurology, Monash Health, Monash University, Clayton, VIC, Australia; ^3^Department of Emergency Medicine, Monash Medical Center, Monash University, Clayton, VIC, Australia; ^4^Department of Pharmacy Monash Medical Center, Monash University, Clayton, VIC, Australia

**Keywords:** stroke, antithrombotics, aspirin, TIA, thrombolysis, partial correlation network, antiplatelet, dysphagia

## Abstract

**Background:** There is emphasis on timely administration of thrombolysis and clot retrieval but not antithrombotic therapy within 48 h for ischemic stroke (frequency of 64% in Australia and 97% in North America). We planned to assess the time metrics and variables associated with delaying antithrombotics (antiplatelet and anticoagulant therapy) administration.

**Methods:** This was a retrospective study at Monash Health over 12 months in 2015. We plotted the cumulative event and mapped the key drivers (dimensionless variable Shapley value/SV) of antithrombotics.

**Results:** There were 42 patients with transient ischemic attack/TIA and 483 with ischemic stroke [mean age was 71.8 ± 15.4; 56.0% male; nil by mouth (NBM) 74.5 and 49.3% of patients received “stat” (immediate and one off) dose antithrombotics]. The median time to imaging for the patients who did not have stroke code activated was 2.3 h (IQR 1.4–3.7), from imaging to dysphagia screen was 14.6 h (IQR 6.2–20.3), and from stopping NBM to antithrombotics was 1.7 h (IQR 0–16.5). TIA patients received antithrombotics earlier than those with ischemic stroke (90.5 vs. 86.5%, *p* = 0.01). Significant variables in regression analysis for time to antithrombotics were time to dysphagia screen (β 0.20 ± 0.03, SV = 3.2), nasogastric tube (β 19.8 ± 5.9, SV = −0.20), Alteplase (β 8.6 ± 3.6, SV = −1.9), stat dose antithrombotic (β −18.9 ± 2.9, SV = −10.8) and stroke code (β −5.9 ± 2.5, SV = 2.8). The partial correlation network showed that the time to antithrombotics increased with delay in dysphagia screen (coefficient = 0.33) and decreased if “stat” dose of antithrombotics was given (coefficient = −0.32).

**Conclusion:** The proportion of patients receiving antithrombotics within 48 h was higher than previously reported in Australia but remained lower than the standard achieved in North American hospitals. Our process map and network analysis show avenues to shorten the time to antithrombotic.

## Introduction

There has beensignificant emphasis on hyperacute therapies for acute ischemic stroke in the advent of endovascular clot retrieval and thrombolytic therapies (1). The components of stroke unit care which have received most attention include swallowing assessment, fever and sugar management (2). Surprisingly, there has been less attention on timely administration of antiplatelet therapy. The role of aspirin in reducing stroke recurrence has been shown in several trials and meta-analyses (3, 4). Importantly, there was reduction in mortality among patients who received antithrombotics within 48 h compared to those having delayed administration (5). Investigators from Get with the Guidelines have set a minimal threshold of 85% as optimal for antithrombotic administration within 48 h. They have demonstrated an improvement in antithrombotic administration within 48 h from 91.5 to 97.0% over 5 years (6), using their performance improvement program. High rates of antithrombotics administration similar to the North American investigators have been described in Taiwan (7) and Korea (8). Elsewhere, national audits in countries, such as New Zealand (21%) (9), United Kingdom (58.6%) (10), Spain (77%) (11), and Australia (71%) (12) have not met this target of 85%. The aim of this study is to assess the time and the relationship among the variables associated with delay to antithrombotic therapy.

## Methods

We retrospectively assessed data relating to time to antithrombotic therapy in patients admitted to the stroke unit at Monash Health over a 12-month period in 2015. Data from this cohort on pneumonia has been published recently (13). In brief, we performed retrospective review of the medical records, extracting demographic data, age and gender, admission diagnoses, time to triage, imaging, and time to administration of antithrombotics, information on dysphagia screen and nil by mouth (NBM) status. Antithrombotics refer to antiplatelet or anticoagulant drugs.

The diagnosis of stroke or transient ischemic attack (TIA) was confirmed by a stroke neurologist and all patients underwent imaging with either CT brain and/or MRI brain. Patients were classified as having a TIA if the neurological deficit lasted <24 h. Patients were classified as having a minor stroke if the National Institute of Stroke Scale (NIHSS) was <5. Time to imaging and time to antithrombotic use was defined as the time from the patient's initial arrival at the emergency department (ED) triage or the documented time of admission to the hospital.

We used the *survival* and *survminer* packages in R (version 3.4.4) to evaluate time to antithrombotic treatment (survival analysis with Kaplan-Meier curve). The *lubridate* package in R was used to parse information on time, enabling analysis of weekday vs. weekend and after-hours effect on time to antithrombotics. Time to antithrombotics after imaging and time to antithrombotics was also analyzed. The regression model was built using available data and excluding variables with high correlation. High correlation occurred between stroke severity at baseline and stroke severity at 24 h, between “stat” (immediate and one off) dose aspirin and stat dose antithrombotics, between Charlson comorbidity index and age of patients; this is understandable as the Charlson comorbidity index (CCI) is a weighted index of comorbid conditions and include age among others in the calculation (14). Due to the high correlations, the variables stroke severity at baseline, stat dose antithrombotics and Charlson comorbidity index were kept in the regression model. This step prevented collinearity in the linear regression analysis. In this analysis, variance inflation factor >10 was considered to indicate presence of multicollinearity (15). To determine the key drivers of the regression model, we calculated the marginal contribution of each covariate to the model or Shapley value (SV) (16, 17). The marginal contribution is determined as the average of all permutations of the coalition of the covariates containing the covariate of interest minus the coalition without the covariate of interest. The positive or negative signs for the dimensionless SV indicate their effect on the direction of the model. This SV analysis was performed using *iml* package in R (17). Plotting was performed using *DiagrammeR/mermaid* (18) and *ggplot2* (19) packages. The network relationships among the variables were explored using Gaussian graphical lasso (20) using *qgraph* package (21). This relationship was created by building a partial correlation network (22). Partial correlation implies a pairwise correlation among the variables after controlling for other variables in a network.

The study was approved by the Monash Health Human Research Ethics Committee. A waiver of individual consent was granted given that the study was retrospective in nature and there was no intervention component to this study.

## Results

Over 12 months in 2015, there were 797 admissions and 617 patients with ischemic (*n* = 525) and hemorrhagic (*n* = 92) stroke. The current analysis is restricted to the 525 patients with TIA (*n* = 42) and ischemic stroke (*n* = 483) in whom both time to antithrombotic therapy and dysphagia screen were available (age 71.8 ± 15.4, male = 56.0%, NIHSS on admission 4 (IQR 2–10). A large proportion, 38.1% (16 of 42) of the patients with TIA and 77.6% (375 of 483) of the patients with ischemic stroke, were kept nil by mouth on admission (NBM). Rectal administration of an antiplatelet (aspirin) was given in 84 patients or 16% of cases (five minor ischemic stroke and 79 ischemic stroke). One of the 13 patients with imaging performed prior to admission was also given rectal aspirin. Aspirin was charted (ordered in medical record) for immediate and one-off (“stat” dose) administration in 42.1% (221 of 525) and given as charted in 89.1% (197 of 221) of cases. Aspirin (*n* = 221) or other antithrombotic drugs (*n* = 38) were charted for immediate (“stat dose”) administration in 49.3% (259 of 525) and given as charted in 86.1% (223 of 259) of cases. The full break down of antithrombotics are described in Table 1. Among patients having antithrombotics charted for immediate administration, there was a significant difference (*p* < 0.01) in the proportion of patients receiving aspirin (197 of 221 or 89.1%) vs. other antithrombotics (26 of 38 or 60.5%).

**Table 1:** Charting of antithrombotics for immediate and one-off “stat” administration.

**Drugs**	**Charted and not given**		**Charted and given**		**Total**
	**As antiplatelet**	**As aspirin**	**As antiplatelet**	**As aspirin**	
Aspirin dipyridamole	1	0	2	1	4
Aspirin	0	23	0	177	200
Aspirin Ticagrelor	0	0	0	5	5
Clopidogrel	11	1	17	11	40
Rivaroxaban	0	0	3	1	4
Warfarin	0	0	4	2	6
Total	12	24	26	197	259

*The term “stat” refers to one-off immediate dosing of medication. Charting of “stat” dose is separate from charting of routine administration of drugs. In a patient admitted at 9 a.m. then charting of aspirin for routine administration at 8 a.m. would mean a waiting time of 23 h. Based on significant difference (p < 0.01) in the proportion of patients receiving aspirin (197 of 221 or 89.1%) vs. other antithrombotics (26 of 38 or 60.5%), we recommend charting of “stat” dose of aspirin in addition to routine charting of “aspirin”*.

### Breakdown of Timelines

**Figure 1:**
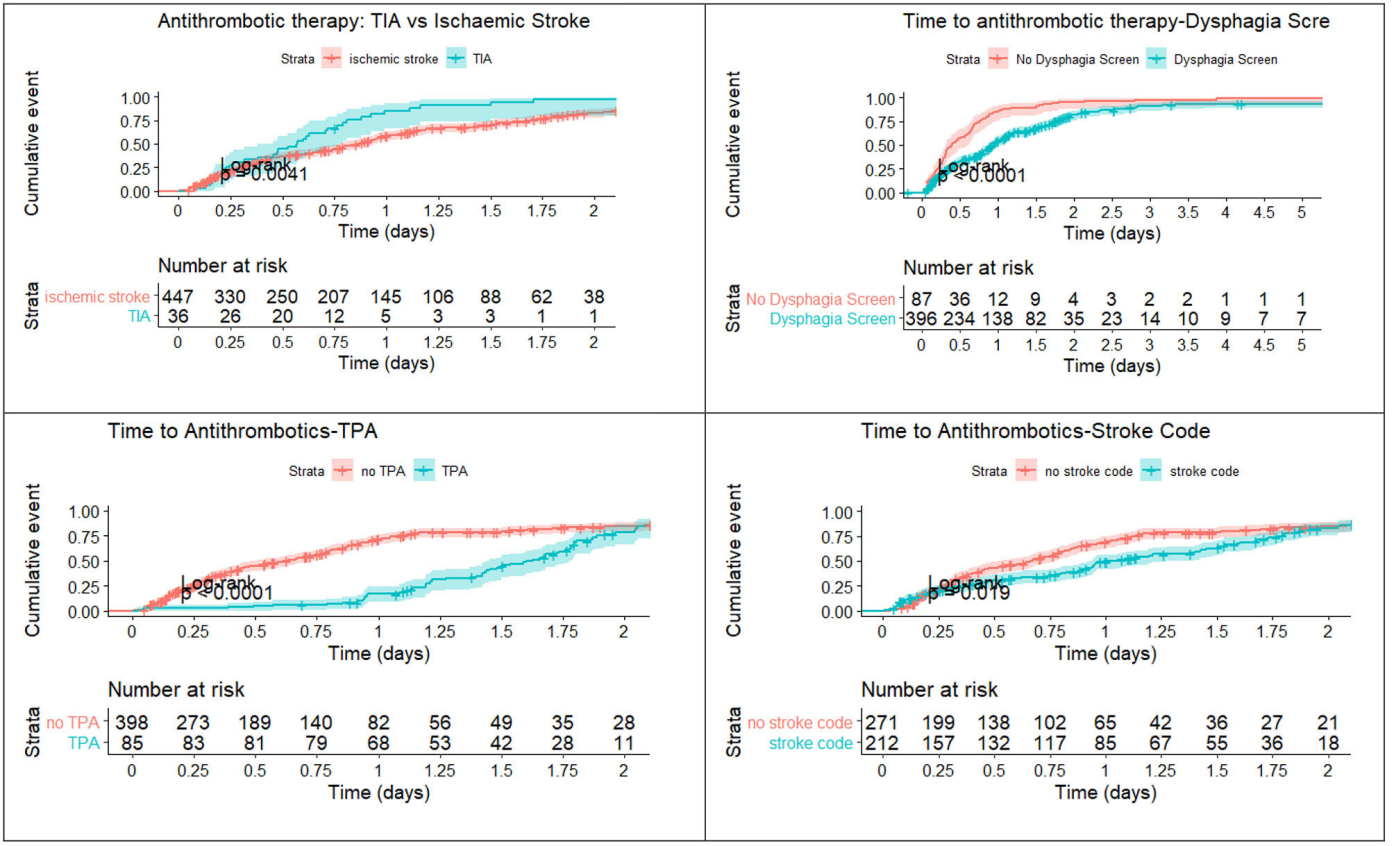
Time to antithrombotics. Patients with TIA received aspirin earlier than those with ischemic stroke. Weekend had no effect on administration of aspirin. The x-axis is truncated at day 2.

The median time from triage to antithrombotic administration **was** 15.1 h (IQR 5.5–27.2). The proportion of patients receiving antithrombotics were 41.7% (*n* = 219) at 12 h, 65.1% at 24 h (*n* = 342), 86.5% at 48 h (*n* = 454), 90.8% at 72 h (*n* = 477), 92.0% at 96 h (*n* = 483), and 93.9% (*n* = 493) at 240 h (Figure 1). Approximately 81.2% (392 of 483) of patients with stroke and 53.8% (seven of 13) patients with scans prior to admission underwent a dysphagia screen. Patients who did not have a dysphagia screen (*n* = 117, mean = 14.9 h) had an earlier time to antithrombotic administration than those who had dysphagia screen (*n* = 408, mean = 23.1 h, *p* < 0.01). There was no statistical difference between weekdays (*n* = 356, mean = 22.6 h) and weekends (*n* = 169, mean = 19.1 h, *p* = 0.88), or office hours from 7 a.m. to 5 p.m. (*n* = 356, mean = 22.6 h) vs. after hours (*n* = 169, mean = 19.1 h, *p* = 0.69) with regards to time to antithrombotics. Stat dose of antithrombotics were charted on the weekend in 45.6% of patients on the weekend and 56.5% of patients on weekday.

**Figure 2:**
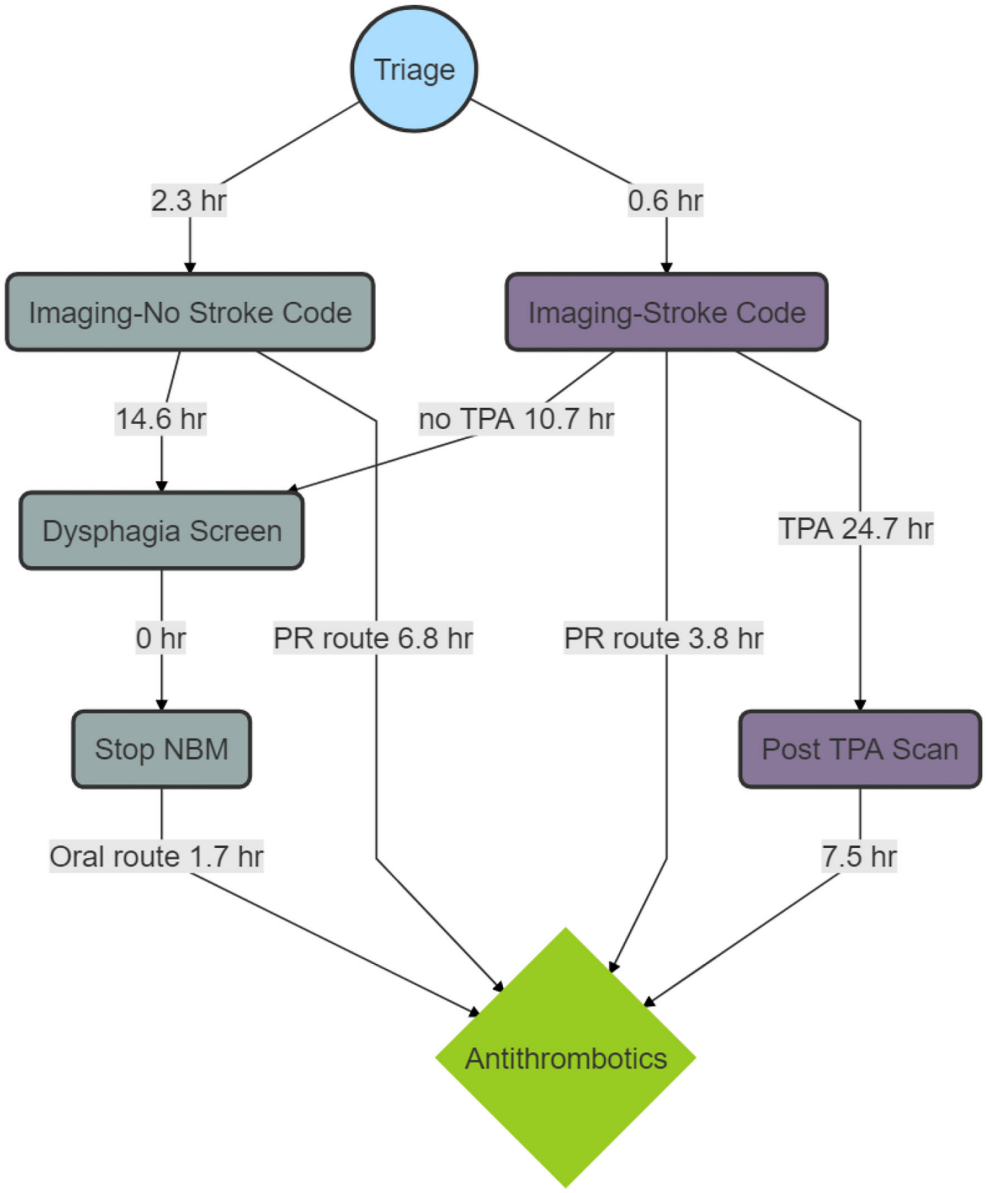
Process map of median time to Antithrombotics. The figure shows a breakdown in timelines. There are delays between imaging and dysphagia screen and between dysphagia screen and stopping nil by mouth status (NBM). The delay between tissue plasminogen activator (TPA) and the post-TPA scan reflects the protocol to delay antithrombotic treatment for 24 h. The delay between the post-TPA scan and antithrombotic administration reflects the delay in assessing the scan and issuing the order for antithrombotics.

A breakdown of the timelines is provided in Figure 2. The median time to imaging for the patients who did not have stroke code was 2.3 h (IQR 1.4–3.7), time from imaging to dysphagia screen was 14.6 h (IQR 6.2–20.3) and time from dysphagia screen to stopping NBM status was 0 h (IQR 0). For the patients who were in the stroke code pathway, the time to imaging was 0.6 h (IQR 0.3–0.9) and the time from imaging to dysphagia screen was 10.7 h (IQR 2.6–18.2). The median time from stopping NBM to antithrombotics was 1.7 h (IQR 0–16.5). For patients who did not have stroke code, the median time from imaging to aspirin by rectal route was 6.8 h (IQR 3.9–12.9) and for those who had stroke code but no alteplase it was 3.8 h (IQR 2.2–12.2). There were 20 patients who had a nasogastric tube (NGT) insertion but the hour date and time information were available in two patients.

### TIA

For patients with TIA, the breakdown in the time to antithrombotic administration is provided in the risk table within Figure 1. Of those with TIA, 45.2% (*n* = 16) received antithrombotic therapy within 12 h, 80.9% (*n* = 34) within 24 h and 90.5% (*n* = 38) at 48 h. Patients with TIA received antithrombotics earlier than those with ischemic stroke (*p* = 0.01, see Figure 1). Approximately 38.1% (16 of 42) of the patients with TIA underwent a dysphagia screen; 16 patients were kept NBM, presumably waiting for a dysphagia screen.

### Alteplase

The median time from alteplase administration to the post-TPA scan was 24.7 h (IQR 23.9–26.0). The median time from the post-alteplase scan to antithrombotics was 7.5 h (IQR 0–17.0). Among patients who had reperfusion therapy, 10 of 88 (11.4%) had the 24-h scan performed during office hours. The baseline NIHSS was 5 (IQR 3–11). After alteplase, the median NIHSS at 24-h was 2 (IQR 0–5).

### Multivariable Regression and Network Analysis

**Figure 3:**
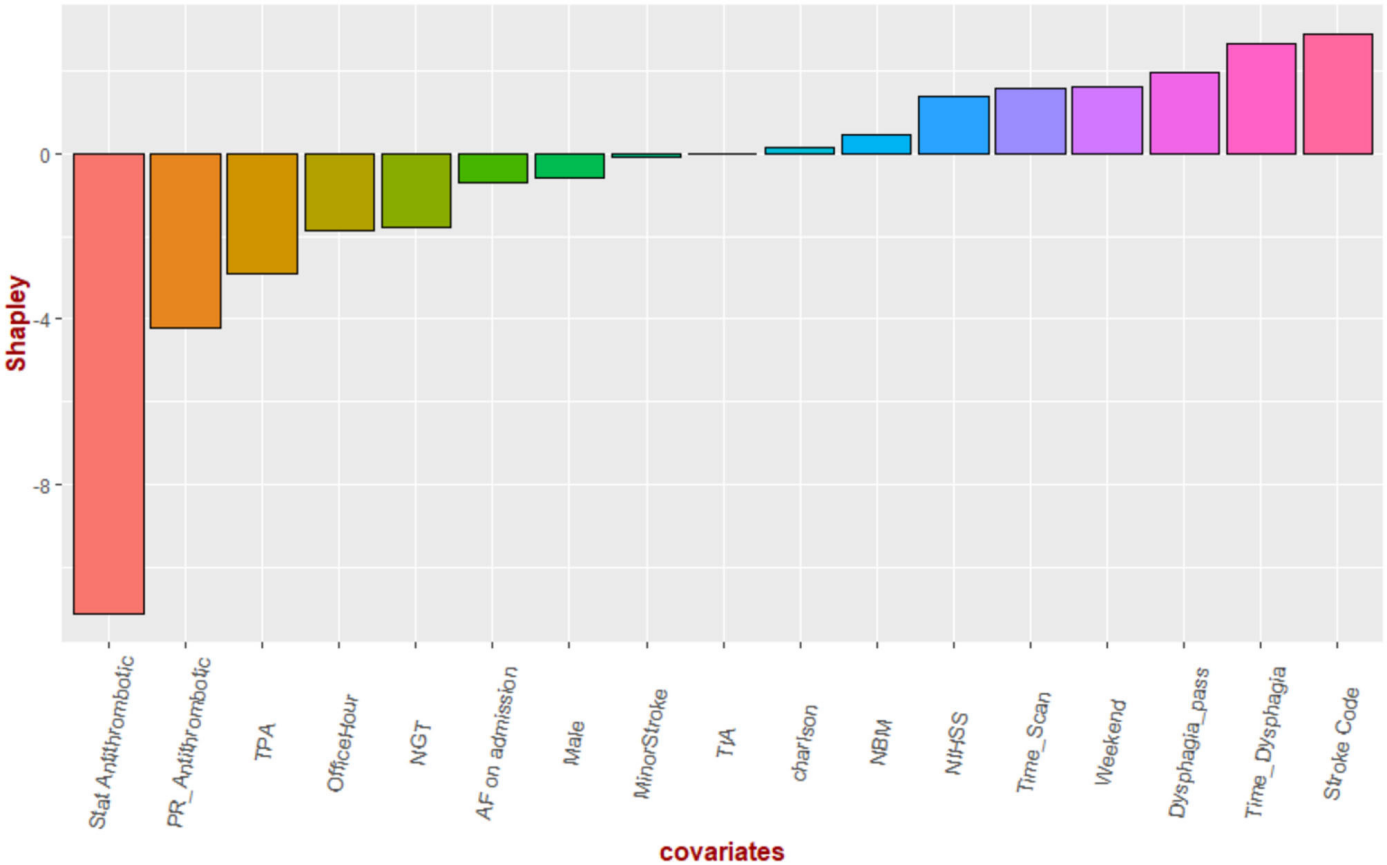
Shapley values. The Shapley values denoting the direction of their effect on time to antithrombotics. Stat dose of antithrombotic had the greatest effect on shortening the time to antithrombotics while delay in dysphagia screen has the opposite effect.

**Figure 4:**
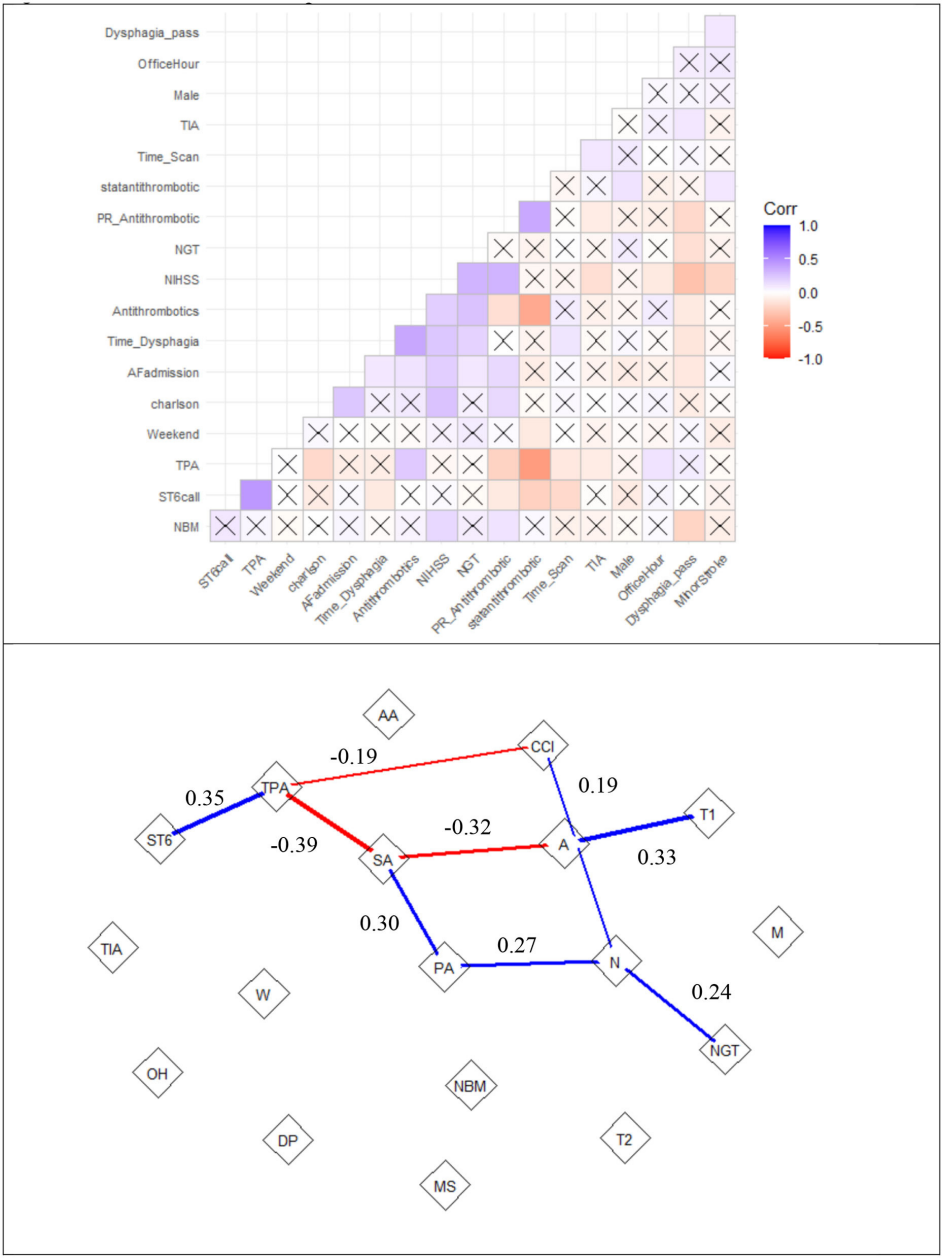
Correlation matrix and partial correlation network of time to antithrombotics. the top row is the correlation matrix. Positive correlations are labeled in blue and negative correlations are in red. The thickness of the line represents the strength of the partial correlation. The “X” represents low correlation (0.0007), such as between *TPA* (alteplase) and *weekend* admission. The bottom row shows the interactions among the variables. The partial correlation network shows relationship between *PR_Antithrombotic* (rectal administration of aspirin or PA) and *statantithrombotic* (“stat” dose of antithrombotics or SA). Note that there a partial correlation of 0.19 between Charlson comorbidity index (CCI) and NIHSS (N) but not with Antithrombotics (A). ST6, Stroke code; AA, Atrial fibrillation on admission; MS, minor stroke; NGT, nasogastric tube; NBM, nil by mouth; DP, dysphagia screen pass; OH, office hour; TIA, transient ischemic attack; T1, time to Dysphagia; T2, time to scan; W, weekend.

In the analysis, all variables had variance inflation factors <1.6, indicating low likelihood of collinearity). Significant variables in multiple regression analysis for time to antithrombotics were time to dysphagia screen (β 0.20 ± 0.03, *p* < 0.01, SV = 3.2), nasogastric tube (β 19.8 ± 5.9, *p* < 0.01, SV = −0.20), TPA (β 8.6 ± 3.6, *p* = 0.01, SV = −1.9), stat dose antithrombotic (β −18.9 ± 2.9, *p* < 0.01, SV = −10.8), and stroke code (β −5.9 ± 2.5, *p* = 0.02, SV = 2.8) (see Figure 3). There was a trend to significance with these variables: stroke severity (*p* = 0.07, SV = 1.3), weekend effect (*p* = 0.12, SV = 1.8) and rectal administration of aspirin (*p* = 0.12, SV = −4.5). The model had an R^2^ value of 0.39. The partial correlation network shows the relationship among the variables (Figure 4). Time to antithrombotics was positively correlated with time with dysphagia screen (partial correlation coefficient 0.33) and negatively correlated with stat dose of antithrombotics (partial correlation coefficient −0.32). In turn, stat dose antithrombotics was negatively correlated with alteplase (partial correlation coefficient −0.38) but positively correlated with rectal administration of aspirin (partial correlation coefficient 0.30). The other connected variables within the partial correlation network were: nasogastric tube insertion, stroke severity and stroke code activation.

## Discussion

The key finding in this study is that the proportion of patients with ischemic stroke or TIA receiving antithrombotics within 48 h was higher than previously reported in Australia but remained lower than the standard achieved in North American hospitals. According to the Shapley value, the variables dominating the regression models were stat dose of antithrombotics, alteplase and time to dysphagia screen. Next, we borrowed an idea from market research (22) to unravel the interactions among key drivers of time to antithrombotics administration. This analysis revealed a possible strategy to shorten the time to antithrombotics, using “stat” dose of antithrombotics rather than wait for the regular time the next day.

We observed that TIA patients received antithrombotics earlier than those with stroke. The reasons behind this might include the presence of dysphagia in patients with ischemic stroke compared to those with TIA. An Australian wide audit found that among admitted TIA patients, the frequency of aspirin within 48 h was 73% (23). In that study, a higher frequency (77%) of patients underwent dysphagia screening. It is possible that the lower frequency of dysphagia screen (38%) might related to shorter time to antithrombotics in our patients with TIA. It is not clear that dysphagia screen is necessary in patients with TIA as these patients have resolution of neurological deficit and low probability of getting pneumonia (13). Furthermore, among patients discharged to an outpatient TIA pathway, the frequency of early aspirin was 92.2% (24). It is interesting to note that it was possible to give aspirin within 24 h of onset in a trial which compared intravenous alteplase to oral aspirin for patients with minor stroke (NIHSS <5) (25). In that study, 39% of the patients had facial palsy and 28% had dysarthria (25). Several trials of aspirin vs. glycoprotein II inhibitors required patients to be given the drug immediately after randomization (26, 27).

A breakdown of the variables in this study show delays between imaging and dysphagia screening and between dysphagia screening and stopping NBM status. This issue possibly reflects the lack of availability of nurses who are trained to perform dysphagia screening in our institution. The Acute Screening of Swallow in Stroke or TIA (ASSIST) tool is used for dysphagia screening of patients in the State of Victoria, Australia (28). This tool uses a combination of variables from other dysphagia screening tools. It is highly sensitive and the presence of facial weakness would result in a patient being deemed to have failed the dysphagia screen. In our institution, patients who have failed this screening test are required to be assessed by a qualified speech therapist. Due to this potential for delayed therapy, 16% of patients were given aspirin by rectal method; five of these patients were classified as having a minor stroke. Various guidelines (National Institute for Health and Care Excellence (NICE) and Canadian Stroke Best Practices) have recommended the use of rectal administration of aspirin or via nasogastric tube in patients with dysphagia (29, 30). An alternative route that has not been explored is the use of intravenous or chewable aspirin. These routes may be more comfortable than the rectal route research in this area is sparse. Further chewable aspirin can disperse in the mouth without water. The cost of chewable aspirin is $AUD 0.15 per tablet, rectal aspirin is $5.30 and intravenous aspirin is $20.00 per dose. Other antiplatelet agents (clopidogrel, combined aspirin and clopidogrel, or aspirin and dipyridamole) can't be given by rectal or intravenous route. These agents may require administration via a nasogastric tube if appropriate, or otherwise experience delay in therapy until dysphagia improves.

The delay between alteplase administration and the post-alteplase scan is understandable and reflects the protocol to delay antithrombotic treatment for 24 h (1). Very early administration of intravenous aspirin has a >3-fold increase risk of intracranial hemorrhage (31). There are recent audits suggesting that the 24-h CT scan can be omitted in stable patients or those with low stroke severity (32, 33). The stroke severity among these patients at 24-h was low, with 75% having an NIHSS of <5. Perhaps one approach is to perform the post-alteplase CT scan to during office hours for these patients and then give aspirin at the 24-h mark. Only a small fraction (11.4%) of our patients had this important scan performed during office hours (7 a.m. to 5 p.m.). The 7.5-h delay between the post-alteplase scan (Figure 2) and antithrombotic administration might reflect the delay in assessing the scan and issuing the order for giving antithrombotics. Less commonly, the finding of intracranial hemorrhage, either as a complication of thrombolysis or as hemorrhagic transformation into the ischemic lesion, may result in consideration of further delay in commencement of secondary prevention.

Our analysis suggested important roles played by “stat” dose of antithrombotics, nasogastric tube insertion and rectal administration of aspirin in reducing the delay (Figure 3). On the other side the use of alteplase, time to dysphagia screen exacerbate delay to antithrombotics. Our search for key drivers were based on coalition game theory to understand the marginal contribution from each covariate in the regression analysis (16, 17). The Shapley value reframes the contribution of NGT to a lesser role than that if it was based on regression coefficient or significant value. However, the identities of key drivers may suggest that these key drivers of the regression model act on their own; its narrow focus do not provide an insight into the interactions among the covariate (22). This issue led us to the idea of combining regression with a network approach to obtain an overall view of the relationship among the key drivers of the model (Figure 4) (22). To do this successfully we had used partial correlation network to gain insight into the chain of covariate linked to our dependent variable (time to antithrombotics). For example, Figure 4 illustrates that the sequence of effect from NGT to stroke severity to rectal use of aspirin and finally “stat” dose use of aspirin. This covariate in turn shorten the time to antithrombotics. It showed that the stroke code variable was related to time to antithrombotics because of its effect on patients receiving TPA; in turn, the use of TPA has a negative effect on time to antithrombotics. In this cohort, nasogastric tube feeding was correlated with time to antithrombotics via its interaction with severe stroke. Our previous assertion about the narrow focus of regression model is that it does not explain how the (significant) covariates are related to the dependent variables. Within this network any of the covariate can be framed as a dependent variable and in turn the network analysis can provide an overview before performing multiple regression analyses.

These analyses suggest a possible strategy to shorten the time to antithrombotics, namely the use of “stat” dose antithrombotics. This strategy was employed in 49.3% of our patients and was given as charted in 86.1% of cases. For example, aspirin is often given at 8 a.m., hence charting of aspirin in a routine manner at 9 a.m. would mean that the patient has to wait nearly 23 h to receive it. Education of staff should target the use of stat dosing of antithrombotics, rather than charting these therapies at routine times. Additionally, medical staff may need to verify that the medication was administered after charting or that charting of the drug is consistent with the patients' oral intake status. For example, a patient may have been charted for aspirin but the medication was not given as the patient was listed as NBM. In this case, improvements could be achieved by ensuring accountability to report the issue to the medical officer so that a different strategy for medication administration could be implemented.

### Limitations

We acknowledge limitations of the study design, including the fact that this was a retrospective, single center study. While a multicenter study may provide a larger sample size, it does not allow the opportunity to analyze time stamped data in details. One issue with the current data is that the time of prescribing antiplatelet drugs is not known, as the prescriber provided a date of signature but was not required to provide a time. Hence delay between the act of prescribing to administration of antithrombotic therapy cannot be determined. It is hoped that once electronic medical record data become available, time stamped data can aid the analysis. The concept of the weekend effect has previously been reported as impacting the quality of care of stroke patients and potentially leading to increased mortality (34, 35). This weekend effect has been postulated to be due to factors, such as lack of medical and allied health staff, and reduced adherence to protocols. This has not been the case in our data and may possibly have been offset by the use of stat dose antithrombotics (see Figure 4 on partial correlation network).

## Conclusion

The proportion of patients with ischemic stroke or TIA receiving antithrombotic therapy within 48 h in our study was higher than previously reported in Australia and New Zealand. Our data are not of the high standard set by North American hospitals (11). The detailed process mapping in this study provide a view into this issue. We hope that by drawing attention to this neglected aspect of acute stroke care, it will improve the time to antithrombotic therapy, and ultimately, clinical outcomes. Finally, our systematic approach can also be applied to other aspects of stroke care.

## Data Availability Statement

The raw data supporting the conclusions of this article will be made available by the authors, upon written request and ethics approval.

## Ethics Statement

The studies involving human participants were reviewed and approved by Monash Health Human Research Ethics Committee. Written informed consent for participation was not required for this study in accordance with the national legislation and the institutional requirements.

## Author Contributions

TP: design and analysis. SS, TK, CM, TP, JL, JV, and AL: data acquisition. TP, BC, SS, TK, CM, HM, JL, JV, and SH: manuscript. All authors contributed to the article and approved the submitted version.

## Conflict of Interest

The authors declare that the research was conducted in the absence of any commercial or financial relationships that could be construed as a potential conflict of interest.
